# Comprehensive Identification and Analyses of the GRF Gene Family in the Whole-Genome of Four Juglandaceae Species

**DOI:** 10.3390/ijms232012663

**Published:** 2022-10-21

**Authors:** Zhongrong Zhang, Shaowen Quan, Jianxin Niu, Caihua Guo, Chao Kang, Jinming Liu, Xing Yuan

**Affiliations:** 1 Department of Horticulture, College of Agriculture, Shihezi University, Shihezi 832003, China; 2 Xinjiang Production and Construction Corps Key Laboratory of Special Fruits and Vegetables Cultivation Physiology and Germplasm Resources Utilization, Shihezi 832003, China

**Keywords:** Juglandaceae, *GRF* gene family, evolutionary analyses, codon bias, expression pattern analysis

## Abstract

The *GRF* gene family plays an important role in plant growth and development as regulators involved in plant hormone signaling and metabolism. However, the Juglandaceae *GRF* gene family remains to be studied. Here, we identified 15, 15, 19, and 20 *GRF* genes in *J. regia*, *C. illinoinensis*, *J. sigillata*, and *J. mandshurica*, respectively. The phylogeny shows that the Juglandaceae family GRF is divided into two subfamilies, the ε-group and the non-ε-group, and that selection pressure analysis did not detect amino acid loci subject to positive selection pressure. In addition, we found that the duplications of the Juglandaceae family *GRF* genes were all segmental duplication events, and a total of 79 orthologous gene pairs and one paralogous homologous gene pair were identified in four Juglandaceae families. The *Ka/KS* ratios between these homologous gene pairs were further analyzed, and the *Ka/KS* values were all less than 1, indicating that purifying selection plays an important role in the evolution of the Juglandaceae family *GRF* genes. The codon bias of genes in the GRF family of Juglandaceae species is weak, and is affected by both natural selection pressure and base mutation, and translation selection plays a dominant role in the mutation pressure in codon usage. Finally, expression analysis showed that *GRF* genes play important roles in pecan embryo development and walnut male and female flower bud development, but with different expression patterns. In conclusion, this study will serve as a rich genetic resource for exploring the molecular mechanisms of flower bud differentiation and embryo development in Juglandaceae. In addition, this is the first study to report the *GRF* gene family in the Juglandaceae family; therefore, our study will provide guidance for future comparative and functional genomic studies of the *GRF* gene family in the Juglandaceae specie.

## 1. Introduction

The Juglandaceae is a family of Dicotyledones Hamamelididae, with about 10 genera and 63 species, and is widely distributed in South and North America, southeastern Europe, East Asia, and Southeast Asia. It is mainly composed of three subfamilies: Engelhardioideae, Juglandaceae, and Rhoipteleoideae [1]. Among them, The Juglandaceae contains commercially important woody trees commonly called walnut, pecan, and hickory. Walnuts (*Juglans regia*), pecans (*Carya illinoinensis*), and iron walnuts (*Juglans sigillata*) are important woody oil trees whose fruits are very high in unsaturated fatty acids and have various health benefits such as blood lipid regulation and immune enhancement [2]. Manchurian walnut (*Juglans mandshurica*) has a variety of industrial uses and medicinal properties and is rich in a variety of biologically active substances such as Juglone (5-hydroxy-1, 4 naphthoquinone) [3].

The General Regulatory Factor (*GRF*) gene family (14-3-3 proteins) is widely present in plants, vertebrates, and higher eukaryotes and is a class of highly conserved acidic proteins with a mass of approximately 30 kDa [4]. It affects changes in target proteins by forming homo- or heterodimers containing nine α-helices in reverse parallel to interact with specific target proteins [5]. *GRF* genes were first isolated from bovine brain tissue and are ubiquitous in eukaryotes, and 14-3-3 proteins can bind to nearly 200 target proteins in a phosphoserine-dependent and phosphoserine-non-dependent manner [6].

The *GRF* gene family plays an important role in plant growth and development. *Arabidopsis* isoforms *14-3-3μ* and *14-3-3ʋ* have delayed flowering under long day conditions [7]. Heterologous expression of the wheat *Ta14-3-3* gene in *Arabidopsis* resulted in stunted plant growth and delayed flowering [8]. In rice, transgenic plants overexpressing *GF14c* had delayed flowering, while the knockout mutant exhibited early flowering [9]. In addition, a flowering activation complex (FAC) in rice consisting of hd3a, 14-3-3 protein, and the bZIP transcription factor OsFD1 is able to activate the expression of *OsMADS15* (the rice homolog of *Arabidopsis APETALA1*), leading to flowering [10]. The 14-3-3 protein is able to interact with oleuropein sterols (BR), abscisic acid (ABA), and jasmonic acid (JA) [11,12]. *AtGRF6* and *AtGRF8*, which negatively regulate the nuclear accumulation of BZR1, are important transcription factors in BR signaling [13]. The 14-3-3 proteins interact with members of the ABA response element binding factor (ABF) family to participate in GA and ABA signaling [14]. The 14-3-3 proteins also play a role in abiotic stress responses, and overexpression of cotton *GF14λ* produces a ‘stay green’ phenotype and increases resistance to stress [15].

So far, the *GRF* gene family members have been identified in most plants, for example, 15, 8, and 21 *GRF* genes have been identified in *Arabidopsis*, rice, and soybean, respectively [16,17,18]. However, no reports on this gene family have been found in the Juglandaceae specie. Therefore, in this study, we identified the members of the *GRF* gene family in the Juglandaceae family on a genome-wide scale and analyzed their gene structures, chromosomal localization, synteny relationships, transcription factor binding sites, expression patterns in flower buds, protein tertiary structures, and phylogenetic relationships, and the results of this study will provide an important basis for the functional resolution of the *GRF* gene family in the Juglandaceae family and its role in flower bud differentiation.

## 2. Results

### 2.1. Identification of GRF Family Genes and Protein Structure Analysis in the Juglandaceae Family

A total of 69 GRF sequences were obtained in four Juglandaceae family species after local blast program comparison and Hmmsearch search, and further confirmed by Batch-CD-search and SMART (Figure S1) tools. They were named based on their genetic position on the chromosomes. The results showed that the mean amino acid lengths of *Jr*GRFs, CiGRFs, JsGRFs, and JmGRFs were 257 aa, 258 aa, 242 aa, and 249 aa, respectively, and the mean molecular weights were between 27.31 kDa (JsGRFs) and 29.13 kDa (CiGRFs), with isoelectric points between 4.78 (CiGRFs) and 5.93 (JsGRFs) (Table S1), suggesting that most of the hoodiaceous GRF proteins are weakly acidic. However, there are some individual ones that are basic, such as JmGRF15. To further analyze the structure of Juglandaceae family GRF proteins, represented by walnut, we used SWISS-MODEL to predict the protein tertiary structure of JrGRF and retained the results with similarity greater than 30%, and found that the JrGRF protein family with similar 3D conformation consists mainly of α-helices with symmetric the spatial structure (Figure S2).

### 2.2. Evolutionary Relationships and Selection Pressure Analysis

**Figure 1:**
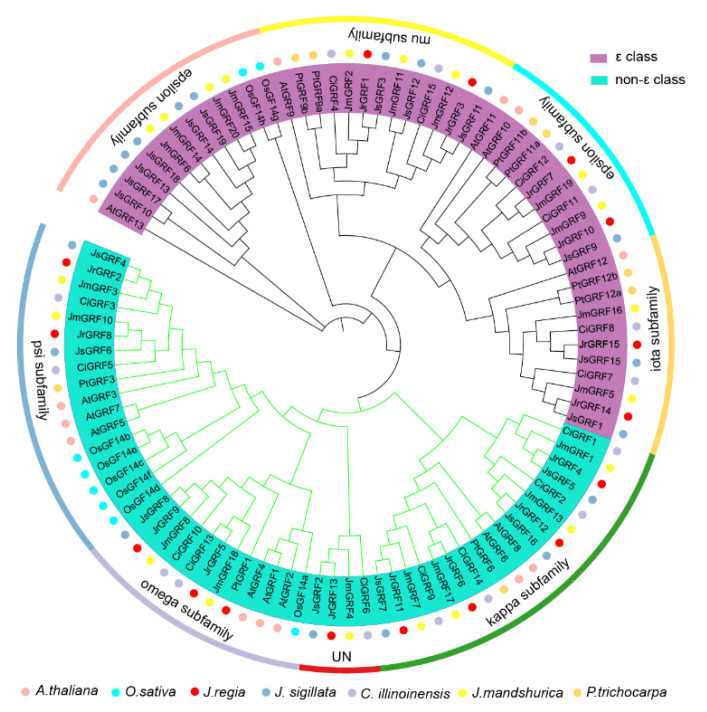
Phylogenetic tree of the *GRF* gene family in the four Juglandaceae species (*J. regia*, *C. illinoinensis*, *J. sigillata*, and *J. mandshurica*) and *O. sativa*, *P. trichocarpa*, and *A. thaliana*.

To study the evolution of the *GRF* gene family in the Juglandaceae family, we selected 99 GRF proteins from seven species, of which 69 sequences were from the Juglandaceae family, 20, 15, 19, and 15 of which were contributed by Manchurian walnut, walnut, iron walnut, and pecan, respectively, and the remaining 30 sequences were from *Arabidopsis* (13), rice (8), and poplar (9). We used MEGA software to construct an evolutionary tree for the GRF family of seven species based on the NJ (neighbor-joining) method. A total of 99 GRF proteins were divided into two major evolutionary branches, 48 in the epsilon evolutionary branch and 51 in the non-epsilon evolutionary branch. The epsilon evolutionary branch was further divided into three sub-evolutionary branches, in which the mu, iota, and epsilon subfamilies contain 13, 11, and 24 GRF members, respectively. The non-ε subfamilies include three groups: omega (12), psi (17), and kappa (18). Notably, the walnut families JsGRF2, JmGRF4, JrGRF13, and CiGRF6 are in the non-epsilon evolutionary branch but are not clustered in any of the subfamily evolutionary branches (Figure 1).

Selection pressure analysis showed that under the site model conditions, each branch was assumed to evolve at the same rate, while each locus evolved at a different rate. the M0 model was used to estimate the baseline ω value, and the GRF family had a small ω value of 0.13123, indicating that purifying selection was the dominant selection. Model M1a showed that 94.41% of loci were conserved with an ω value of 0.12795. In addition, Models M2a, M3, and M8 also had ω values less than 1, and no amino acid loci subject to positive selection pressure were detected (Table S2).

### 2.3. Gene Structure and Conserved Motif Analyses

**Figure 2:**
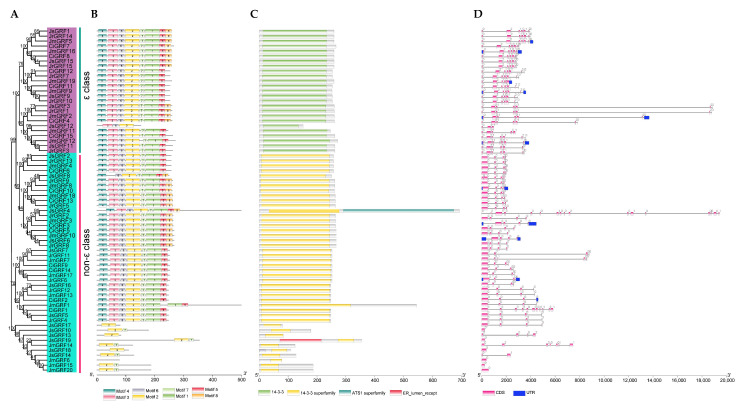
Phylogeny (**A**), motif analysis (**B**), main domain (**C**), and gene structure (**D**) of each *GRF* gene and GRF protein. Exons, introns, and upstream/downstream are represented by red, black, and blue lines, respectively.

Four conserved structural domain types were detected in 69 GRF proteins from four Juglandaceae species, in which all members of the ε group contained 14-3-3 domains, the non-ε group contained 14-3-3 superfamily domains, and some also contained other domains, such as JsGRF4, which also contained ATS1 superfamily domains (involved in cell cycle control, cell division, chromosome assignment, and cytoskeleton composition-related families) (Figure 2B). A total of eight unique motif patterns were observed. Among the members of each subgroup, motif patterns were mostly similar and consistent between the ε and non-ε groups, but some members of the non-ε group showed considerable differences, containing only Motif1 and Motif2 (Figure 2A). The exon/intron structure showed that the vast majority of members of the non-ε group of the pecan GRF family contained introns 1-4, while the ε group contained introns 1-6, with significant differences in exon/intron patterns between the two groups (Figure 2C).

### 2.4. Chromosomal Distribution and Synteny Analysis of GRFs

**Figure 3:**
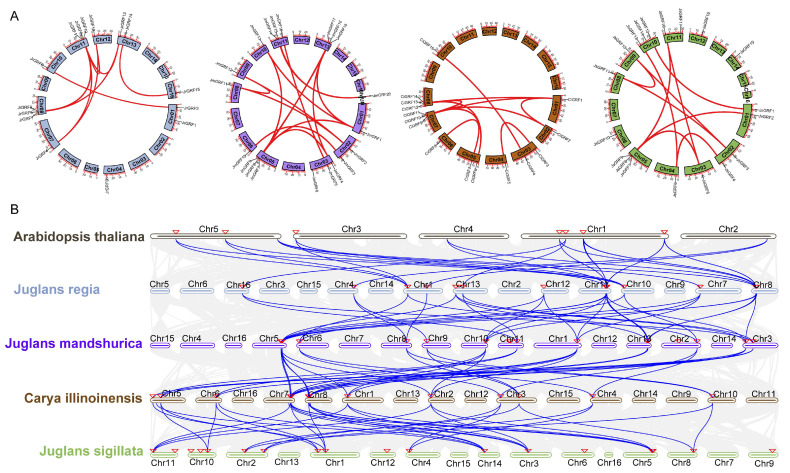
(**A**) Collinearity of segmental duplication gene pairs of *GRFs* in four Juglandaceae species. (**B**) Collinearity between *Arabidopsis thaliana* and four Juglandaceae *GRF* gene families. The red lines represent the segment duplication (SD) gene pairs of the *GRFs*.

The chromosomal localization results showed that the 15 *JrGRF* genes were localized on 9 of the 16 chromosomes, with a higher distribution on chromosome 8 and chromosome 11, containing three members, while the pecan and Manchurian walnut GRF family genes were mainly distributed on chromosomes 3, 5, 7, 8, 10, 11, and 13. (Figure 2A). In addition, the vast majority of Juglandaceae family *GRF* genes are distributed on both ends of the chromosomes. Gene duplication plays an important role in the expansion of plant and animal gene families, including tandem and fragment duplication [19]. Therefore, potential duplication events in the Juglandaceae family GRF were analyzed. In total, 10 gene pairs of *JrGRFs* (13 genes) were located in the segmental duplication region, accounting for 86.7% of the number of *JrGRFs* (Figure 3A). In the pecan, all *Ci*GRF family members underwent segmental duplication, forming a total of 12 gene pairs. Overall, 19 members were detected in Manchurian walnut, forming 16 gene pairs, accounting for 95% of the *JmGRFs*, and 13 members were detected in iron walnut with fragment replication events, forming 10 gene pairs, accounting for 86.7% of the iron walnut *GRFs*. No tandem duplication events were detected in any of these four Juglandaceae species. These findings suggest that segmental duplication is a major factor contributing to the expansion of *GRF* gene families in the Juglandaceae family, especially in pecan and Manchurian walnut.

To further explore the synteny relationships between the walnut *GRF* gene family and other species, we constructed synteny maps between walnut, *Arabidopsis* and three species of the Juglandaceae family, including *J. regia* vs. *A. thaliana*, *J. regia* vs. *C. illinoinensis*, *J. regia* vs. *J. mandshurica*, and *J. regia* vs. *J. sigillata* (Figure 3B). There were 25, 37, 37, and 29 homologous gene pairs, respectively. Syntenic events were concentrated on chromosomes 5, 8, 11, and 13.

To further investigate the expansion and contraction of the GRF family in Juglandaceae species, we analyzed the loss and duplication events of GRF family genes in four Juglandaceae species using Notung software. The results showed five gene losses, four gene duplications, and 11 gene co-divergences in the common ancestor of walnut, Manchurian walnut, and iron walnut. In the Juglandaceae family, there were 11 gene duplications and 2 gene losses. We found that the GRF family underwent expansion in the Manchurian walnut when compared to walnut and iron walnut, with more *GRF* gene duplications in Manchurian walnut (+3) than walnut (+0) and iron walnut (+1), but more gene losses in Manchurian walnut (−5) than in walnut (−2) and iron walnut (−1). In conclusion, all the GRF families of the Juglandaceae family underwent gene loss events during evolution (Figure S3).

### 2.5. Identification of Orthologous and Paralogous GRF Family Genes

**Figure 4:**
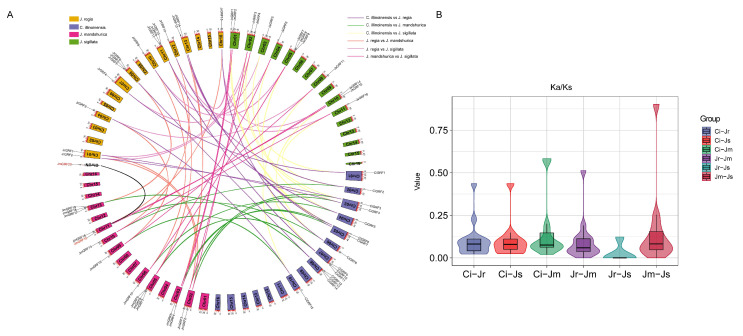
(**A**) Circle plot of paralogous and orthologous *GRF* gene pairs among the four Juglandaceae species. The number on the scale represents the physical location of each chromosome. (**B**) *Ks* and *Ka/Ks* values of orthologous *GRF* gene pairs between any two of the four Juglandaceae species.

To further explore the relationship between the GRF family genes of walnut, Manchurian walnut, iron walnut, and pecan, orthologs and paralogs between them were identified separately (Figure 4A, Table S3). A total of 79 pairs of orthologous genes and one pair of paralogous genes (*JmGRF15*-*JmGRF20*) were identified in six combinations, with the largest number of homologous genes between walnut and pecan (15). In addition, the number of homologous gene pairs between walnut, pecan, and Manchurian walnut was 14, and the number of homologous genes between walnut, pecan, Manchurian walnut, and iron walnut was 12 (Table S3). In addition, most of these homologous genes were distributed on chromosomes 1, 2, 5, 7, 8, 10, and 13 in our four Juglandaceae family species (Figure 4A). In addition, we further analyzed the *Ka/KS* ratios between these homologous gene pairs and all *Ka/KS* values were less than 1, indicating that the *GRF* genes of the Juglandaceae family are mainly influenced by purifying selection in the evolution (Figure 4B).

### 2.6. Prediction of Cis-Acting Elements in the Promoter of the GRF Gene of the Four Juglandaceae Species

**Figure 5:**
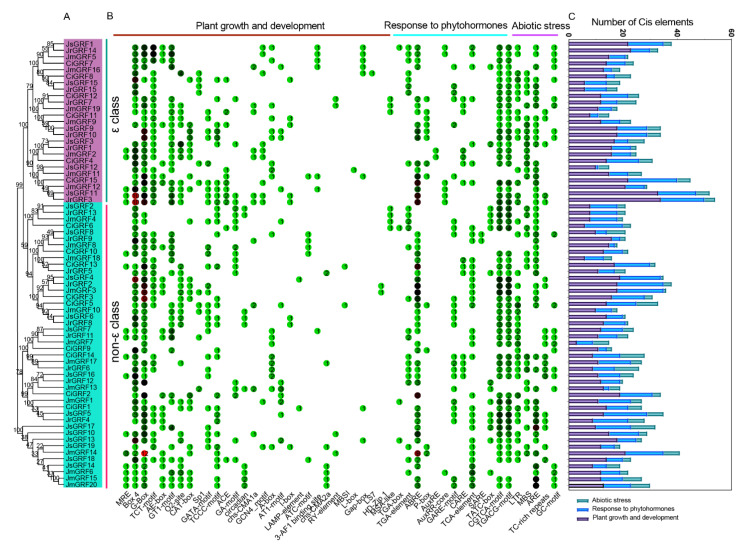
Phylogenetic relationship (**A**), distribution (**B**), and number (**C**) of cis-elements in the promoter region of *GRFs* of Juglandaceae. PlantCARE identified cis-acting elements using the 2000 bp upstream sequence of the Juglandaceae *GRF* gene.

To identify cis-acting elements in the promoter of the Juglandaceae family *GRF* gene, the 2.0-kb sequence upstream of the translation start site was analyzed. The cis-acting elements were divided into three categories: growth and developmental responses (e.g., cell cycle regulation, meristematic tissue expression, circadian rhythm control, endosperm expression, etc.), phytohormone responses (e.g., JA, growth hormone, GA, ABA, SA), and adversity stress elements (e.g., drought, low temperature, defense elements, etc.) (Figure 5, Table S4). The phytohormone-acting elements are mainly involved in the abscisic acid, growth hormone, gibberellin, jasmonic acid (JA), and salicylic acid pathways. Among these elements, the abscisic acid response element (ABRE) was the most abundant, followed by the jasmonic acid action elements (CGTCA-motif and TGACG-motif). Light responsive elements (Box 4 and G-Box) were the most abundant acting elements in the growth and development response group. Low temperature (LTR) and anaerobic inducible elements (ARE) were present in most of the Juglandaceae family *GRF* genes. The above results suggest that *GRF* genes may play an important role in low temperature as well as in the regulation of ABA and SA.

### 2.7. Codon Bias Analysis of the Juglandaceae GRF Gene Family

We extracted CDS sequences from the GRF family of four Juglandaceae species. We also checked the length of the coding regions of all GRF family members using FasParser software, removing members with sequences less than 300 bp and ensuring that they all used ATG as the start codon and TAA, TAG, or TGA as the stop codon. Immediately afterwards, we performed a series of codon bias indices, including ENC (effective number of codon), where the smaller the value, the stronger the codon bias. The ENc values of the GRF family genes in Manchurian walnut, pecan, walnut, and iron walnut were 43.37–60.88, 41.65–61, 41.89–60.74, and 44.72–60.74, respectively, with mean values of 51.91, 51.082, 51.867, and 52.301, respectively. The closer the CAI (codon adaptation index) was to 1, the stronger the codon bias. Manchurian walnut, pecan, walnut, and iron walnut were 0.17–0.297, 0.214–0.316, 0.215–0.312, and 0.165–0.299, respectively, with mean values of 0.239, 0.256, 0.252, and 0.234, respectively. The CBI (codon bias index) values, responding to the components of highly expressed superior codons in a gene, for the *GRF* gene family of Manchurian walnut, pecan, walnut, and iron walnut, were in the range of −0.108–0.136, −0.095–−0.121, −0.1–0.164 and −0.1–0.15. The values of FOP (frequency of optical codons) ranged from 0 to 1, with 1 indicating that only the optimal codons were used and 0 indicating that no optimal codons were used. The FOP (frequency of optical codons) ranged from 0 to 1, with 1 indicating that only optimal codons were used and 0 indicating that no optimal codons were used. The mean values of FOP of the four Juglandaceae species ranged from 0.429, 0.443, 0.438, and 0.427, indicating that both optimal and nonoptimal codons were used in the four Juglandaceae species, and in conclusion, the codon bias of the GRF family genes in Juglandaceaes was weak (Table S5).

**Figure 6:**
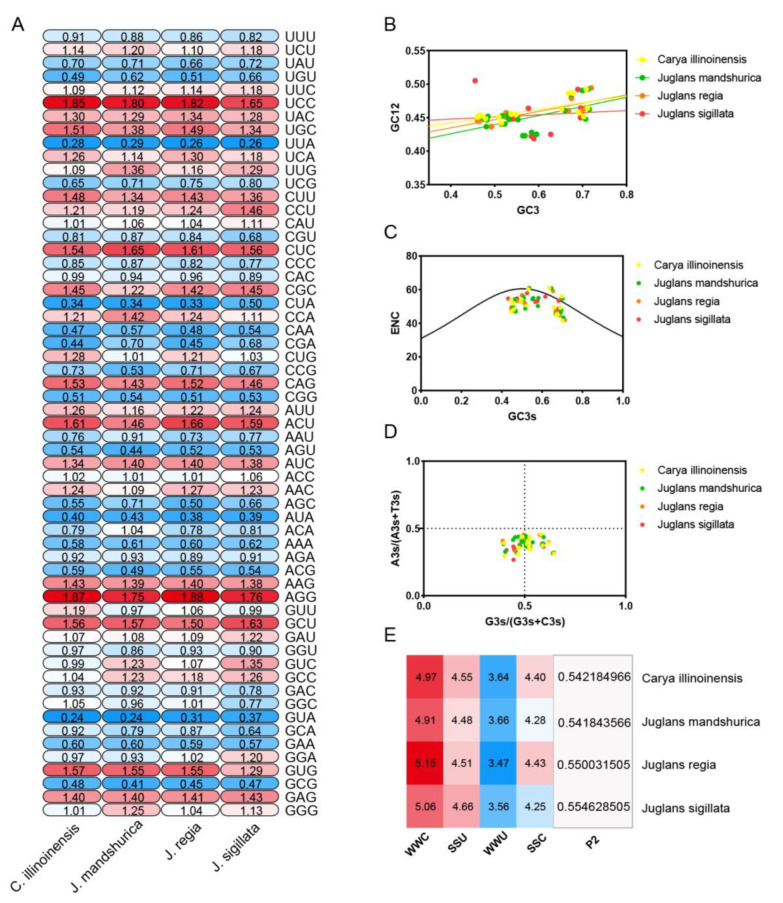
Codon bias (**A**), ENC-plot (**B**), Neutrality-plot (**C**), PR2-plot (**D**), and P2 analysis (**E**) in four Juglandaceae *GRF* gene families.

We further investigated the ENc-GC3s, Neutrality-plot, PR2-bias plot, and P2 (translational selection index) of the Juglandaceae family *GRF* genes and found that most members of the GRF family genes were distributed far below the standard curve, and individual members were distributed near the standard curve and the standard curve, indicating that Natural selection pressure and base mutations jointly influenced the codon preference of the *GRF* gene family in Juglandaceae (Figure 6B). Neutrality-plot analysis showed significant positive correlations between GC12 and GC3 in walnut, pecan, and Manchurian walnut with *p* values of 0.002, 0.007, and 0.012, respectively, and codon usage was influenced by mutations. The correlation between GC12 and GC3 content of iron walnut was not significant (*p*-value = 0.65), indicating that the base composition of positions 1, 2, and 3 are different, the genomic GC content is highly conserved and the codon use is more influenced by selection (Figure 6B). The PR2-bias plot results showed a distribution in the lower left and lower right, indicating that the GRF family genes of the Juglandaceae family are uneven for the use of A, C, G and T (Figure 6D). The P2 (translational selection index) values were positively correlated with gene level expression [20]. All members of the Juglandaceae GRF family have P2 values greater than 0.5, which indicates that translational selection plays a dominant role in codon usage for mutational pressure (Figure 6E).

RSCU, relative synonymous codon usage, provides a visual indication of the preference of codon usage [21]. If there is no preference for codon usage, the RSCU value of the codon is equal to 1. When the RSCU value of a codon is greater than 1, it indicates a relatively high frequency of usage. The results showed that there were 31, 32, 30 and 33 codons with RSCU > 1 in pecan, Manchurian walnut, walnut, and iron walnut, respectively. In addition, four Juglandaceae family species had a strong preference for four codons: CUC, AGG, UCC, and ACU (RSCU > 1.6) (Figure 6A).

### 2.8. Gene Expression Analysis of GRF Family in Juglandaceae

**Figure 7:**
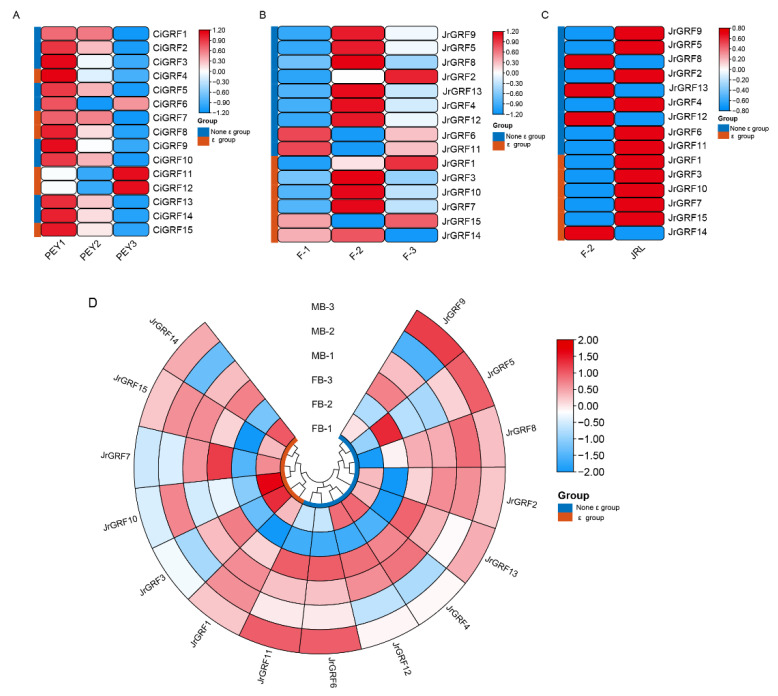
Expression patterns of walnut *GRF* genes (*CiGRFs* and *JrGRFs*) in flower buds and embryos. (**A**) Expression patterns of *CiGRFs* in embryonic development. (**B**) Expression patterns of *JrGRFs* in female flower bud differentiation of *Juglans regia* cv. Xinxin No.2. (**C**) *JrGRFs* expression in female flower buds and leaf buds of *Juglans regia* cv. Xinxin No.2. (**D**) Expression of *JrGRFs* in male and female flower buds in *Juglans regia* L. cv. Wen185.

To reveal the expression patterns of the Juglandaceae *GRF* gene families in floral bud differentiation as well as embryo development, we used transcriptome data from pecan embryos at different developmental stages, female and leaf buds of *Juglans regia cv. Xinxin No.2*, and female and male floral buds of *Juglans regia L. cv. Wen185*. The vast majority of pecan GRF genes were highly expressed in early pecan embryo formation and gradually decreased as the embryo developed, including *CiGRF3/4/8/9/10/13/14/15*. Notably, *CiGRF11/12* were mainly highly expressed in late embryo development and lowly expressed in the middle stage (Figure 7A). The results showed that nine *JrGRF* genes (i.e., *GRF3/4/5/7/8/9/10/12/13*) were highly expressed at the F-2 stage of floral bud differentiation and lowly expressed at the early stage of floral bud differentiation. In contrast, *JrGRF1* and *JrGRF2* were highly expressed during the F-2 period of floral bud differentiation (Figure 7B). In both flower buds and leaf buds of ‘Xinxin No.2‘, all *JrGRF* genes were highly expressed in leaf buds except for *GRF8/12/13/14* (Figure 7C). The genes highly expressed in the FB-1 period of female floral bud differentiation in ‘Wen 185‘were *JrGRF3/4/5/10/12*, in addition to *JrGRF6*, *JrGRF9*, and *JrGRF11*, which were mainly expressed in the MB-3 period of male floral bud differentiation (Figure 7D). In conclusion, the above results suggest that *JrGRF* and *CiGRF* genes play important roles in floral bud differentiation and embryo development (Figure 7, Table S6).

### 2.9. Prediction of Walnut GRF Gene Transcription Factor Binding Sites

**Figure 8:**
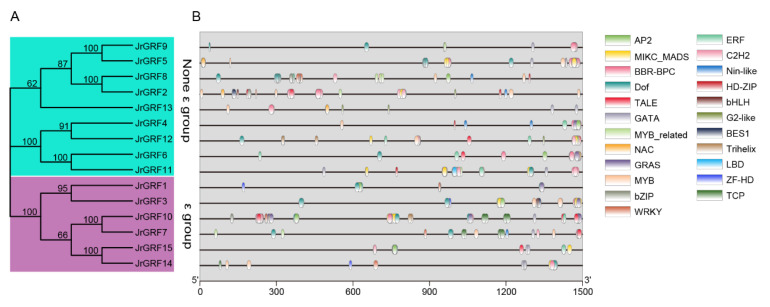
Analysis of *JrGRF* gene transcription factor binding sites. Phylogenetic relationship (**A**), and Transcription factor binding sites of *JrGRFs* (**B**).

Transcription factor binding sites (TFBSs) are vital regulatory components of gene transcription in promoters. Most genes conserved among species are usually shown to be conserved in their TFBS. To further investigate the transcription factor binding sites of the *GRF* gene family in the walnut, plant TFDB was used to make predictions of TFBSs within the promoter regions. The results showed that TFBSs in the promoter regions of different *GRF* genes differed significantly in type and number. A total of 676 binding sites of 23 transcription factors were identified. Among them, *JrGRF9* contained the lowest type and number of transcription factors (19 binding sites for 7 transcription factors), and *JrGRF2* contained the highest type and number of transcription factors (154 binding sites for 17 transcription factors) (Figure 8).

### 2.10. Expression Analysis of JrGRF Gene in Different Tissues and Different Stages of Female Flower Bud Differentiation

**Figure 9:**
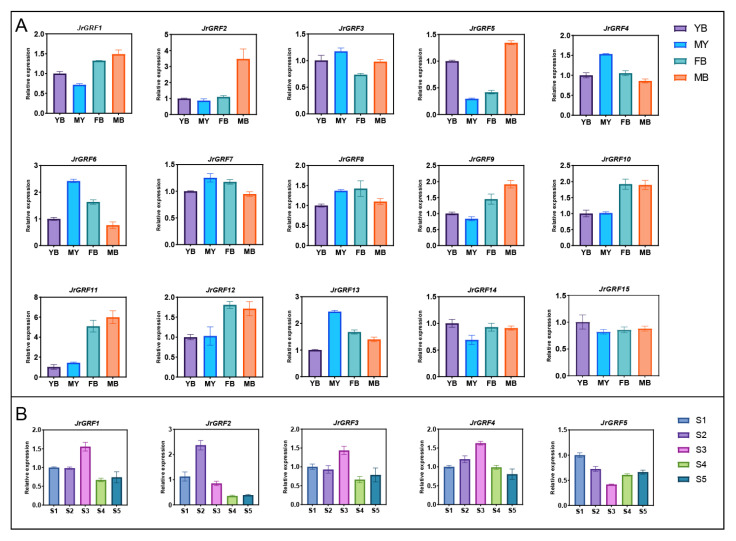
Relative expression of *JrGRF* in different tissues and stages of female flower bud differentiation. (**A**) The expression pattern of *JrGRFs* in different tissues of walnut. (**B**) Expression pattern of *JrGRFs* in female flower bud differentiation of walnut.

To investigate the expression of *JrGRF* genes in different tissues, qRT-PCR analysis was conducted on leaf buds, leaves, female flower buds and male flower buds samples collected from ‘Xinxin No.2’ using the gene expression of walnut leaf buds as a control. The results showed that the expression of *JrGRF14* and *JrGRF15* was higher in ‘Xinxin No.2’ leaves than in other tissues. *JrGRF2* and *JrGRF9* were mainly highly expressed in male flower buds, and the genes that were highly expressed in both female and male flower buds were *JrGRF1/9/10/11/12*, while *JrGRF4* and *JrGRF6* were highly expressed mainly in mature leaves, and the above results indicated that the expression of *JrGRF* genes in ‘Xinxin No.2’ was tissue-specific (Figure 9A). Flower bud differentiation is an important process in plant flowering, the role of walnut GRF genes and flowering genes in walnut flowering bud differentiation was further investigated. The expression of the first collected sample (S1) was used as a control, and the expression of *JrGRF1/3/4* was found to be higher in S3 than in other developmental periods. In addition, the *JrGRF2* gene was highly expressed mainly during S2 and *JrGRF1* during S1 (Figure 9B).

## 3. Discussion

The *GRF* gene family members play important roles in plant growth and development by interacting with target proteins related to plant hormone signaling and physiological metabolism. We performed a comprehensive and systematic analysis of the *GRF* gene family in the Juglandaceae family using bioinformatics methods for phylogeny, covariance, gene structure, motifs, structural domains, protein structure, chromosomal location, codon preference, and expression analysis for four species of the Juglandaceae family.

### 3.1. Identification, Evolution and Gene Structure Analysis of GRF Gene Family

**Figure 10:**
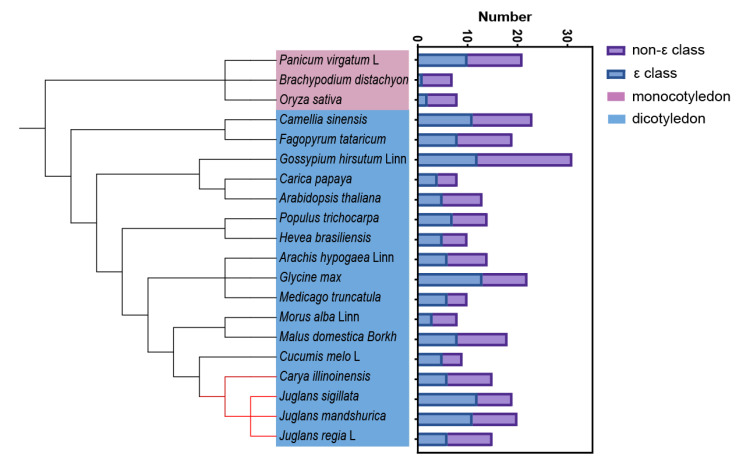
Comparison of the number of *GRF* genes in 20 crops.

Based on the reported *GRF* genes, we counted a total of 306 *GRF* genes from 20 studied species, 36 in monocotyledons, and 216 in dicotyledons, with the largest number of *GRF* genes containing dicotyledons in monocotyledons and the largest number of 31 in dicotyledons in land cotton. To further analyze the differences in the number of *GRF* genes in these species, population classification was performed and *GRF* genes were divided into two groups (non-ε and ε groups) (Figure 10). The number of non-ε groups was higher than ε groups in all monocotyledons, and three results were observed in dicotyledons, with non-ε groups > ε groups, such as *Juglans regia* L., *Carya illinoinensis*, and *Arabidopsis thaliana*, non-ε groups = ε groups and non-ε groups < ε groups in *Populus trichocarpa*, *Carica papaya*, and *Hevea brasiliensis*, such as *Juglans mandshurica*, *Juglans sigillata*, *Cucumis melo* L., and *Medicago truncatula*. These results suggest that the pecan *GRF* gene family evolved in different plants through gene loss and increase and thus to better adapt to survival [22,23,24,25,26,27,28,29,30,31,32,33,34]. Physicochemical analysis showed that the majority of Juglandaceae family GRF proteins are acidic, similar to studies in papayas and poplars [31,33]. In addition, the Juglandaceae GRF protein family triple structure consists mainly of α-helices, and the phylogenetic relationships of Juglandaceae *GRF* gene family members can be classified into ε-like and non-ε-like classes, with no amino acid loci detected that are subject to positive selection pressure. Gene structure analysis showed that ε-like *GRF* genes contain more exons and introns than non-ε-like *GRF* genes, suggesting that this evolution has driven this diversity, and the above results are similar to those of the mango 14-3-3 family of proteins [35]. The vast majority of the Juglandaceae GRF family have similar and consistent motif patterns between the ε and non-ε groups, but some members of the non-ε group show considerable variation, containing only Motif1 and Motif2, which may be related to the evolution of individual Juglandaceae species.

### 3.2. Analysis of GRF Gene Duplication, Collinearity, and Codon Bias

Gene duplication events play an important role in gene family expansion and may have gained functional diversity during evolution [36]. Segmental duplication occurs in 48 gene pairs in the GRF family of Juglandaceae, while no tandem duplication events occur, suggesting that fragment duplication may be the main driver of expansion of this gene family. Since the beginning of comparative genomics, it has been assumed that direct homologs retain their function better than paralogs [37,38]. Therefore, we calculated *Ka/Ks* values for 79 pairs of orthologs and one pair of paralogs, with *Ka/Ks* values less than 1, indicating that they evolved mainly under strong purifying selection, which is similar to the results of studies in soybean [18]. The promoters of the pecan *GRF* gene family contain multiple light response elements, which suggests that *GRF* genes are induced by light signals and involved in complex light signal responses. In *Arabidopsis*, the 14-3-3 protein (Phot1) specifically binds under blue [39] and red light, and *At*14-3-3μ and *At*14-3-3ν interact with photoperiodic regulatory proteins. In addition, we also identified many hormone-related cis-regulatory elements, including abscisic acid, salicylic acid, growth hormone, and gibberellin corresponding factors. The 14-3-3 proteins not only enhance the stability of *Arabidopsis ABF* genes, but also are part of the ABA-regulated gene transcription complex, which together constitute the rice VP1 (the basic leucine zipper transcription factor EmBP1 and Viviparus1) complex with a role in binding the ABA response element Em1a [40,41]. We also identified binding sites for many important transcription factors, NAC [42], ERF [43], bHLH (Basic/Helix-loop-Helix) [44], among other TFBSs associated with plant growth and development, these different TFBSs may provide valuable clues for studying how plants affect growth and development by regulating the spatiotemporal expression of Juglandaceae 14-3-3. The weak codon preference of Juglandaceae family *GRF* genes is influenced by a combination of natural selection pressure and base mutations, and translational selection plays a dominant role in codon usage against mutational pressure, which is largely consistent with our previous studies within the walnut bZIP family [45].

### 3.3. GRF Gene Expression Analysis

We investigated the expression patterns of *CiGRF* genes in pecan embryogenesis, which can be divided into two major categories based on expression, one with high expression in early embryogenesis, such as *CiGRF3* and *CiGRF4*, and one with high expression in late embryogenesis, such as *CiGRF11* and *CiGRF12*. *JrGRFs* have different expression patterns in different tissues of walnuts. *JrGRF9* is an ortholog of *At14-3-3Ω*, which acts as a linker in *Arabidopsis thaliana,* OXS2 can alter the subcellular distribution of FT and indirectly interact with the flower-forming element FT and the transcription factor FD to regulate flowering [46]. In tobacco and *Arabidopsis*, 14-3-3 protein prevents RSGs from entering the nucleus and negatively regulates signaling pathways related to endogenous GA synthesis [47,48]. *OsGF14c* overexpression plants exhibited delayed flowering and the knockout mutants showed early flowering relative to wild-type plants [9]. In our study, *JrGRF2*, is an ortholog of *OsGF14c*, was highly expressed in F-3 and leaf buds (JRL) of ‘Xinxin No.2’ walnut floral bud differentiation, low in FB-2 period of female floral bud differentiation in ‘Wen 185’. The above results suggest that *JrGRF2* may be involved in the differentiation of flower buds. We further investigated the expression trends of the JrGRF gene family in different tissues and at different periods of female flower bud differentiation using qRT-PCR, and the results indicated that the *JrGRF* gene family is tissue-specific and period-specific. For example, *JrGRF2* and *JrGRF9* were mainly highly expressed in male flower buds, while *JrGRF4* and *JrGRF6* were mainly expressed in mature leaves; *JrGRF1* was highly expressed in early female flower bud differentiation, while *JrGRF1/3/4* was highly expressed in the female flower bud differentiation S3 period.

## 4. Materials and Methods

### 4.1. Plant Materials

Based on the observation of the morphological changes in female flower buds before, during, and after the critical period between physiological differentiation and morphological differentiation through paraffin section and freehand slice in the Aksu region of Xinjiang [49], three trees (*Juglans regia* cv. Xinxin No. 2) of the same age, tree condition, and cultivation management level were selected and randomly mixed. The leaves, leaf buds, male flower bud, and female flower bud were collected in the east, west, south, and outer parts of the tree from 31 May to 10 June 2021, once every 2 days. All tissues were immediately frozen in liquid N and stored at −80 until use.

### 4.2. Identification of Juglandaceae Family GRF Gene Family Members

The GRF protein sequences of *Arabidopsis* were downloaded from the TAIR database (https://www.arabidopsis.org/, accessed on 10 August 2022), walnut and pecan whole genome data were obtained from the NCBI database (http://www.ncbi.nlm.nih.gov/, accessed on 10 August 2022), Manchurian walnut whole genome data were obtained from the NGDC database (https://ngdc.cncb.ac.cn/, accessed on 10 August 2022), and iron walnut whole genome data were obtained from the GIGADB database (http://gigadb.org/dataset/100693, accessed on 10 August 2022). The hidden Markov model file (PF00244) for the GRF gene family was downloaded from the pfam data (http://pfam.xfam.org/, accessed 10 July 2022), and then the walnut protein sequences (E ≤ 1 × 10^10^) were searched in hmmer 3.0 software and the results obtained were de-duplicated by SMART (http://smart.embl-heidelberg.de/, accessed on 10 August 2022) and NCBI-CDD (https://www.ncbi.nlm.nih.gov/Struc-ture/cdd/wrpsb.cgi, accessed on 10 August 2022) databases to extract the results for further identification and screening, and finally obtained the walnut GRF family protein sequences [50]. Finally, the primary structure of the Juglandaceae family GRF protein sequences was analyzed using ProtParam (https://www.expasy.org/protparam/, accessed on 10 August 2022) [51], and the online tool SWISS MODEL (https://swissmodel.expasy.org/, accessed on 10 May 2022) to analyze the tertiary structure of walnut GRF proteins [52].

### 4.3. Construction of Phylogenetic Tree and Selection Pressure Analysis

MEGA 7 was used to construct the evolutionary tree using the following parameters, neighbor joining method (NJ), Poisson correction, pairwise deletion, bootstrap value of 1000 other parameters were set as default [53]. The CDS (coding sequences) of four Juglandaceae family species were downloaded and their stop codons were removed and compared using MEGA 7. After saving these files, phylogenetic trees were constructed using the neighbor-joining method (NJ) and tree files without branch lengths were saved. The Seqformat Convertor program in EasyCodeML software was used to convert the multiple sequence comparison files into paml format and perform selection pressure analysis, and in preset mode, site models were selected for positive selection site analysis [54].

### 4.4. Conserved Motifs, Gene Structure and Domain Analysis

GSDS v2.0 (http://gsds.cbi.pku.edu.cn/, accessed on 12 August 2022) was used to predict the intron and exon structures of the *GRF* gene family in the Walnut family [55]. Conserved motif composition parameters of GRF proteins were analyzed using MEME (http://meme-suite.org/tools/meme, accessed on 12 August 2022) using default values [56].

### 4.5. Synteny Analysis of GRF Gene Family

The synteny relationship between walnut and four other species (*Arabidopsis*, Manchurian walnut, pecan, and iron walnut) was searched for using MCScanX [57] and the results were visualized using the ‘Advanced Circos’ program of TBtools [58]. Notung v2.9 software was used to analyze the duplication and loss of GRF family genes [59].

### 4.6. Ortholog, Paralog, and Ka/Ks Calculation

Orthologs and paralogs of the GRF gene family in the Juglandaceae family were determined using OrthoVenn2 (https://orthovenn2.bioinfotoolkits.net/, accessed on 12 August 2022) with e values of 1e-5 [60]. In order to analyze and detect the pattern of gene duplication in the four Juglandaceae species, the non-synonymous (*Ka*) and synonymous substitutions (*Ks*), and evolutionary rates (*Ka/Ks*) in their paralogs and orthologs, were evaluated [58].

### 4.7. Prediction of Promoter Elements

The *GRF* gene promoter region (upstream 2000 bp) was extracted from the whole genome database of the Juglandaceae family and the type, number, and function of the cis-acting elements of the *GRF* gene promoter were analyzed using the PlantCARE website (http://bioinformatics.psb.ugent.be/webtools/plantcare/html/, accessed on 12 August 2022) [61].

### 4.8. Analysis of Codon Preferences in the GRF Gene Family of Juglandaceae Family

The sequences GC, GC1, GC2, and GC3 were analyzed using the Calculation of parameters program [62] from the CAIcal online website (http://genomes.urv.es/CAIcal/, accessed on 23 August 2022), with GC representing the average GC content of codons, GC1, GC2, and GC3 represent the GC content of the first, second and third bases of the triplet codon, respectively, GC12 represents the average GC1 and GC2 values, and GC3 values reflect the codon preference influencing factors (selection pressure and mutation pressure), and the more concentrated the distribution range is, the more influenced by natural selection pressure [63]. The codonW program (version 1.4.2) (http://codonw.sourceforge.net/, accessed on 23 August 2022) was used to calculate the effective codon number (ENC), relative synonymous codon usage (RSCU), the G + C content of the third position of the synonymous codon (GC3s), and the T, C, A, and G content of the third position of the synonymous codon (T3s, C3s, A3s, G3s). RSCU > 1 indicates that the codon is used more frequently, and vice versa lower, with no preference at RSCU = 1.

### 4.9. Expression Analysis of the GRF Gene Family in the Juglandaceae Family

Transcriptome expression data were collected for three different stages of pecan embryo development (http://www.juglandaceae.net/, accessed on 12 July 2022), ‘Xinxin No.2’ leaf buds (JRL) and flower buds (F-1, F-2, F-3), and ‘Wen 185’ male and female flower buds at different stages of differentiation (FB/MB-1, FB/MB-2, FB/MB-3) [49,64].

### 4.10. Transcription Factor Binding Sites of JrGRF Genes

The promoter region (1500 bp upstream) of the walnut GRF family genes was obtained from the genome-wide database. PlantTFDB (http://planttfdb.gao-lab.org/, accessed 10 July 2022) was used to analyze the transcription factor binding sites of the GRF family genes [65]. Finally, the results were visualized using the program ‘Simple BiosequenceViewer’ of TBtools [58].

### 4.11. Gene Expression Analysis

Total RNA was extracted from different tissues and female flower buds at different times using Plant RNA Extraction Mini Kit (Aidlab, Beijing, China) and detected by Thermo Nano Drop 2000 instrument; total RNA was extracted using HyperScriptTM III RT SuperMix for qPCR with gDNA Remover Kit to reverse transcribe into cDNA (TaKaRa, Beijing, China). Real-time fluorescent quantitative PCR was performed with the CFX Connect ™ real-time PCR system (BioRad, Hercules, CA, USA) using 18S as the internal reference gene [66], and all operations were performed according to the instructions. Experiments were set up with three biological weights and three technical replicates. Data analysis of the relative expression of each gene member was performed using 2^−∆∆CT^ [67] and the primer sequences are in Table S7.

## 5. Conclusions

In our study, we identified a total of 69 *GRF* genes in four Juglandaceae species and analyzed their gene structure, domains, and conserved motifs. The phylogenetic tree showed that the Juglandaceae family *GRF* genes were classified into two subgroups: non-epsilon and epsilon. By promoter analysis, *GRF* genes may play an important role in low temperature as well as in the regulation of ABA and SA. The codon preference of GRF family genes in pecan is Juglandaceae and is affected by a combination of natural selection pressure and base mutations, and translational selection plays a dominant role in codon usage in response to mutational pressure. In addition, we analyzed the expression patterns of pecans and walnuts in embryonic development and floral bud differentiation. In conclusion, our study provides a rich genetic resource for exploring the molecular mechanisms of flower bud differentiation and embryonic development in Juglandaceae, and also provides guidance for future comparative and functional genomic studies of the *GRF* gene family in Juglandaceae.

## Data Availability

Not applicable.

## Supplementary Materials

The supporting information can be downloaded at: https://www.mdpi.com/article/10.3390/ijms232012663/s1.
