# Supplementary material for: Comprehensive Identification and Analyses of the GRF Gene Family in the Whole-Genome of Four Juglandaceae Species
Source: Int J Mol Sci. 2022 Oct 21;23(20):12663. doi: 10.3390/ijms232012663 (PMC9604165; doi:10.3390/ijms232012663)
Supplement: Supplementary file 1 [file ijms-23-12663-s001.zip › Figure S3.pdf]

Table Duplication, loss of the GRF gene family in four Juglandaceae species

Species	Duplications	cD_Nodes	Losses
Magnoliopsida	10	0	0
Arabidopsis thaliana	6	0	4
Juglandaceae	11	0	2
Carya illinoinensis	0	0	5
Juglans	4	11	5
Juglans regia	0	0	2
Juglans sigillata	1	0	1
Juglans mandshurica	3	0	5

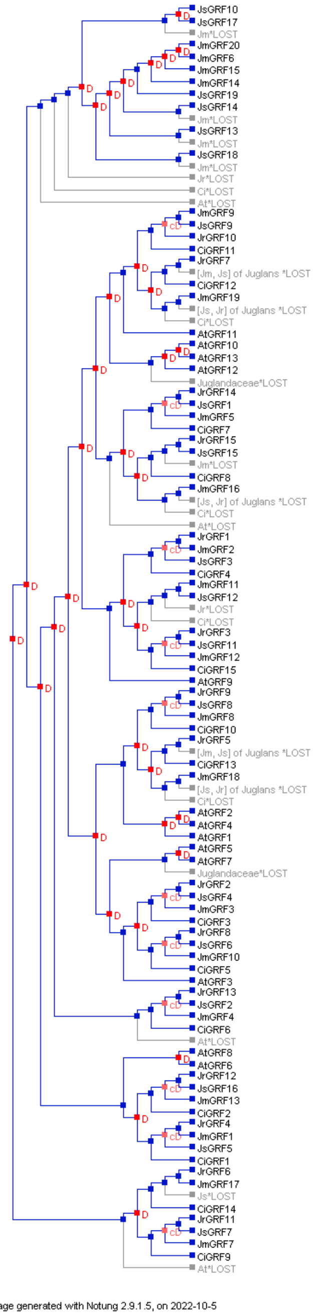

Figure S3 Duplication, loss of the GRF gene family in four Juglandaceae species
