# Supplementary figures and images for: Comprehensive Identification and Analyses of the GRF Gene Family in the Whole-Genome of Four Juglandaceae Species
Source: Int J Mol Sci. 2022 Oct 21;23(20):12663. doi: 10.3390/ijms232012663 (PMC9604165; doi:10.3390/ijms232012663)

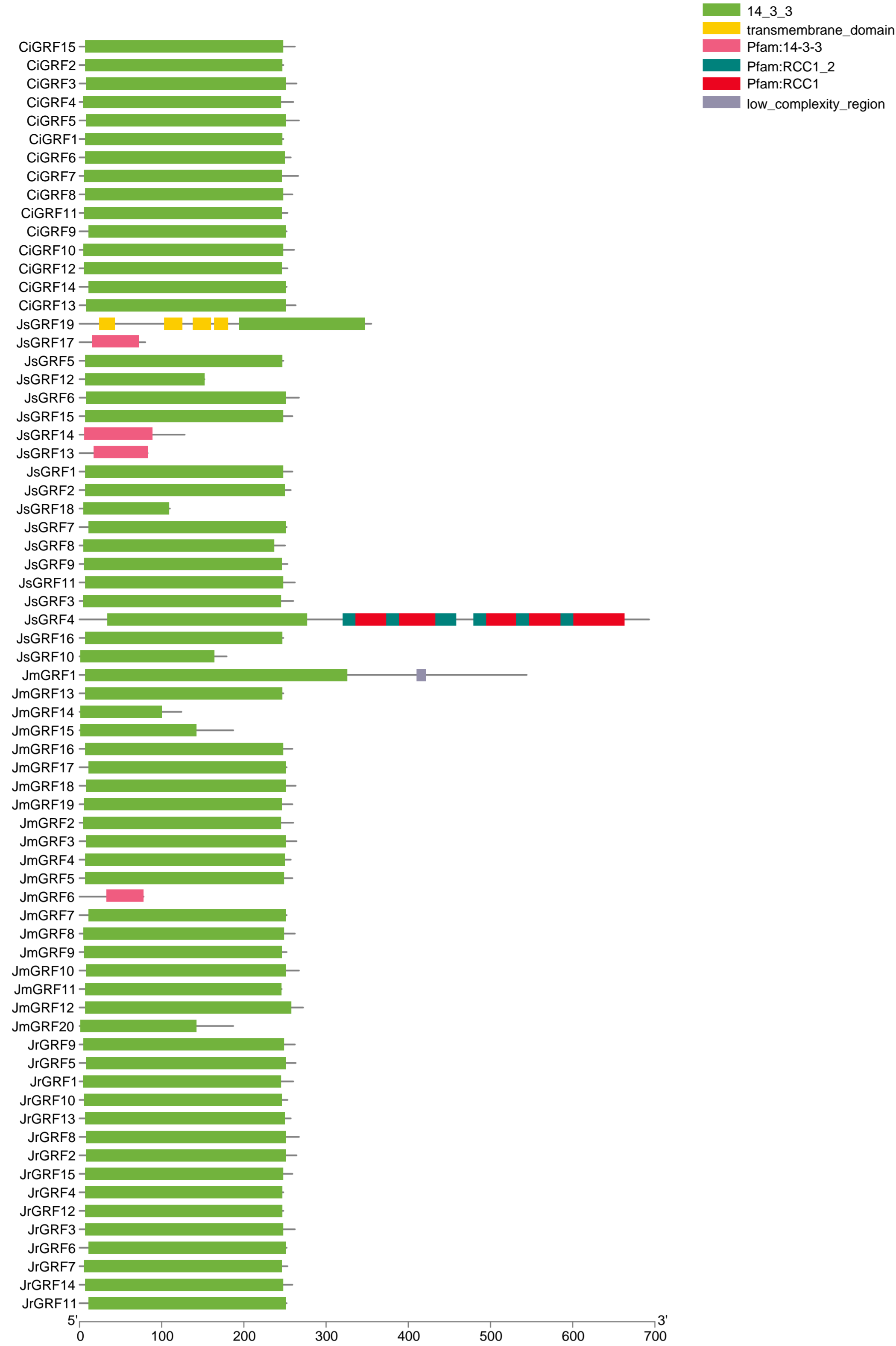

Supplement: Supplementary file 1 [file ijms-23-12663-s001.zip › Figure S1.pdf]

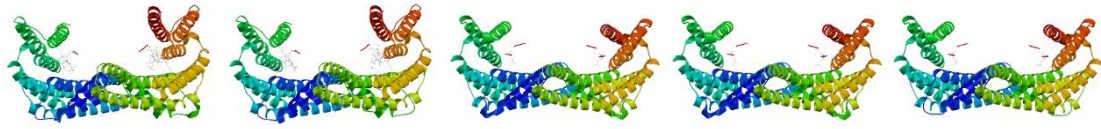

JrGRF9

JrGRF5

JrGRF8

JrGRF2

JrGRF13

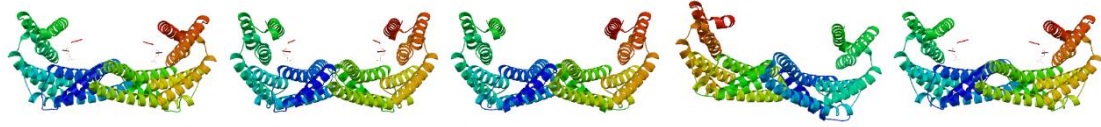

JrGRF4

JrGRF12

JrGRF6

JrGRF11

JrGRF15

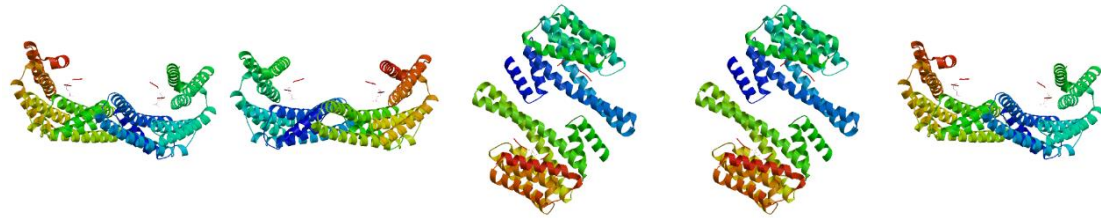

JrGRF1

JrGRF14

JrGRF3

JrGRF7

JrGRF10

Supplement: Supplementary file 1 [file ijms-23-12663-s001.zip › Figure S2.pdf]
